# Are there bubbles in the vanilla price?

**DOI:** 10.1186/s40100-022-00213-y

**Published:** 2022-04-04

**Authors:** Khalid Khan, Chi-Wei Su, Adnan Khurshid, Muhammad Umar

**Affiliations:** 1 School of Finance, Qilu University of Technology, Jinan, China; 2 School of Economics, Qingdao University, Qingdao, China; 3 School of Economics and Management, Zhejiang Normal University, Jinhua, China

**Keywords:** Vanilla price, Vanillin, Cyclone, Price speculation, Bubbles

## Abstract

This paper investigates the presence of the bubbles that are experienced in the global vanilla (VNL) price, using the GSADF approach. The results show that there are five bubbles in the VNL price that are driven by specific reasons. Also, in this regard, the opening and ending points of each bubble coincide with specific events that contribute toward the formation, as well as the rupture of the bubbles. It has also been noted that the cyclone Hudah and the monopoly of the cartels trigger the first bubble, while the regulation and export taxation policy drive the second bubble. However, market-oriented policies, the abolition of cartels, and the exchange rate adjustments are the leading factors that form the third bubble. Furthermore, political instability, hurricanes and bad weather are the key factors driving the fourth bubble. And finally, the rising global demand and decreasing supply, price speculation, poor quality, and cyclone Enawo create the last bubble. It needs the VNL market to be more stable in order to continue supply, which can then control the price fluctuations. The minimum role of the cartels and middlemen is vital for VNL price stability. Therefore, the governments should ideally facilitate the big companies to directly negotiate with farmers which may be beneficial for both companies and the farmers alike.

## Introduction

The purpose of this study is to probe whether the price of vanilla (VNL) experiences the phenomenon of bubbles in addition to this, the study also evaluates why VNL is excessively expensive, and how its price has gone through significant changes over time. Vanilla has a wide variety of uses in the food industry. These uses usually include its inclusion in ice creams, sweet foods, alcohol, cosmetics, and perfumes (Menz and Fleming 1989). It must be noted that vanilla belongs to an industry that is small and its production is quite low, which makes it a product that is not adequate for global consumption. The vanilla (VNL) market involves a few major producing countries such as Madagascar (Zhu 2018), and while the consumers are many, this is exactly what may lead to the difference between the demand and supply, which ultimately exerts pressure on the VNL price. This has then led to the creation of artificial VNL as a substitute for natural VNL, primarily due to the rising costs associated with the pure form. On the other hand, the usage of artificial vanilla may have potentially led to health issues, due to which the companies have now started to turn back to natural forms of VNL. Moreover, it must also be noted that the VNL crop is cultivated in a specific region of the sub-Saharan constituency, fraught with environmental, political, economic and security issues (Wexler 2017; Neimark et al. 2019) which have played a decisive role in the VNL price changes, and lead to a boom and bust. Hence, the phenomenon of the VNL price bubble is a crucial standpoint for policymakers and analysts. The price bubbles and their bursting have previously had a profound impact on the economy and have been studied and analyzed extensively (Khan et al. 2020). The bubble usually occurs when there is a successive rise and abrupt collapse of the prices in a definite period (Lind 2009; Khan et al. 2021). Data tell us that the prices are not consistent in their intrinsic value, which is affirmed to be the most essential example and the most powerful assumption behind the conventional finance theories (Khan et al. 2020). That is to say that if the assets form a price bubble, these assets are then more attractive to investors, and therefore, their demand increases, and the asset prices rise even further. The bubble will, however, disappear if the buyers believe that the prices are no longer rising, which will then reduce demand and may trigger a crisis (Case and Shiller 2003; Shiller 2003). Hence, it can be affirmed that the price bubbles have an essential influence on the financial and real markets and are worthy of study.

Vanilla is the most expensive agricultural product after saffron (Menz and Fleming 1989), as it is quite difficult and delicate to grow. It is the most popularly used in ice creams, sweet foods, alcohol, cosmetics and perfumes. Madagascar is the biggest producer of VNL in the world, and for a century or so, it mostly monopolized the market of vanilla production. Other countries such as Papua New Guinea, India and Uganda are the other major producers of vanilla. On the other side of the spectrum, The USA is the biggest importer of vanilla, primarily due to the significantly big ice cream industry that it hosts. Similarly, the European Union (EU) and China are also among the largest importers of VNL (Cadot et al. 2009). In general, it is common knowledge that the price fluctuations affect agricultural commodities, but VNL is exceptionally volatile due to the liberalization of the market (Turner 2015). Moreover, as this is a small and exclusive industry, even a single hurricane can devastate the entire crop. Also, it takes three years to fully mature, and the flower blooms only for one day. Therefore, in order to produce these precious beans, the crop must be pollinated by hand on the very same day (Neimark et al. 2019). The complete process of VNL cultivation is time consuming and laborious. The growers also have to struggle with thieves who are targeting the VNL crop. In order to avoid these problems, some of the farmers harvest the beans before the plant even matures, but this produces poor-quality VNL which ultimately drives down the price (Neimark et al. 2019). Hence, the deteriorating quality and high prices have an adverse effect on the VNL price bubbles burst. Likewise, climate change is also a phenomenon that is increasing the frequency and intensity of storms in Madagascar, and the farmers are now facing the risk of their crops being destroyed by severe weather events (Pagano and Echevarria 2018). This is the epitome of instability, not only because of the weather, but also due to the issue that the workers themselves are marginalized, a factor due to which there is high susceptibility to disruptions in the market and price. The changes in the market structure are related to the record high prices of VNL, and the various policy changes that have ultimately triggered the price fluctuations (Neimark et al. 2019). Also, the market is now held by cheap artificial VNL since the 1980s to counter the natural VNL, and therefore, the farmers have decreased VNL cultivation as a result of low returns from this industry (Menz and Fleming 1989).

Artificial VNL is derived from synthetic flavoring that is extracted from wood and even petroleum called vanillin. This is a viable substitute to natural VNL and is economical (Cadot et al. 2009). It is expected that vanillin will be adopted more widely in various industries, so as to escape the increasing costs of natural VNL. The demand for natural VNL experienced a rise in 2011, and this is when the companies pledged to exclude artificial from their products and increase the content of natural VNL. Following this, it was expected that the demand for VNL may decline, owing to the impact of terrorist attacks, the global oil crisis and the political crisis, which would then lead to a reduction in the consumer income and VNL price (Danwatch 2018). The product VNL is considered to be physically, socially and politically insecure, and hence, it has been experiencing price fluctuations. It is noteworthy that the price has fluctuated sharply since the market has opened in the year 1989, from $400 per kilogram in 2003 to $30 per kilogram in 2005, a phase that lasted for a decade. Similarly, the VNL price also shot up and became even more expensive than silver. The international price of the bean fell from a high in 2003 to a historical low. However, it was also observed that the VNL price burst and dropped to its lowest level between the years pertaining to 2005 and early 2014, as caused by the abundance of VNL production (Danwatch 2018). This then prompted the farmers extracting vanilla to switch to other crops, primarily because the yields were not enough for them to survive, which eventually lead to shortages. It was noted that the price of VNL was $80 a kilogram in the year 2014, and reached up to a staggering level of $600 in the year 2017 due to its high global demand. Moreover, the companies also shunned artificial VNL in favor of the natural and organic varieties of VNL. Simultaneously, the government then made efforts to regulate the VNL price and industries; wherein, the prices were negotiated at the point of sale, which made the market freer and more volatile (Neimark et al. 2019). In this regard, it was commonly believed that the power to determine the price on the VNL market was mainly with the buyer and the farmer has no way to control supply or withhold the quantity of VNL, therefore, this leads to the increase in the prices. It was in the year 2018 that this bubble burst, as the companies resorted to the use of vanillin due to the high price of natural VNL. Likewise, the bumper crop in the other regions also leads to a rapid decline in the price of VNL. This wide fluctuation in the VNL market has not been considered as a good sign. This is because it may have caused overinvestment into VNL when the price was higher and underinvestment when the price was lower, which is a phenomenon that ultimately leads to price volatility in any industry. Similarly, a greater level of stability is essential for farmers to continue producing a certain crop in the long run as well.

We can thus derive the main contribution of this study in the existing literature in the following forms. First, the investigation of the VNL price has been undertaken, which has scant studies available on the discipline of price bubbles. The VNL crop is a considerably attention worthy crop due to its extensive usage in the food industry, and its production in poverty-stricken and unstable regions can critically disrupt its global supply. Thus, it is crucial to examine the VNL price bubbles in the VNL market, and hence acknowledge this as one of the first studies to evaluate the price bubble phenomenon that is experienced in the VNL market. Second, the study aims to determine the causes of the development of the price bubbles in the VNL market, a concept that has been overlooked in the extant literature established thus far. Therefore, it thoroughly discusses the leading factors, such as the sudden climate changes, political uncertainty, speculation about low quality, development and availability of synthetic VNL and theft, factors which may have contributed to and continue to contribute toward the disturbance of the demand and supply, as well as the price bubble. Lastly, the econometric methodology, such as the Supremum Augmented Dickey–Fuller (SADF) and the Generalized Supremum Augmented Dickey–Fuller (GSADF), has been employed in this study in order to detect the VNL price bubbles. It is noteworthy that this method possesses superior conduct as compared to the other conventional procedures, which can be applied to determine bubbles in any data with varying frequencies. Moreover, these two tests may detect the bubbles in the full and sub-sample as well which can thus offer more convincing results. The study concludes that the VNL prices experience this bubble behavior and therefore detects five bubbles over a period of time. In this regard, the opening point and the ending coincide with specific events that eventually lead to the formation, as well as the puncture of the bubbles. Thus, the mismatch between the global demand and supply, cyclones and other natural disasters, political instability, cartels monopoly, strict government regulation, poor weather, exchange rate fluctuations, theft, price speculation and low quality are some of the leading determinants of the VNL bubbles formation and perforation. The VNL price is also vulnerable to variable supply, uncertain weather and market speculation. Therefore, a greater level of stability is required in the VNL market in order to ensure a continuous supply, which can lead to the control of the usual price volatility. Moreover, the governments should also facilitate the big organizations and companies to directly negotiate with the framers, which may be ultimately beneficial for both the companies and the farmers. Therefore, the government should take measures to overcome the theft problem and can thus enact and levy special laws in order to discourage early harvest of the VNL crop. These huge fluctuations lead to price speculation and unpredictability regarding the price and quality of VNL. Thus, stability is an important component for all the stakeholders in the market. Similarly, the market structure should be devised in such a way that it involves all the participants so as to avoid instability and uncertainty.

We have organized this study into seven sections. The relevant review of the literature has been listed in section “Literature review.” Then, the theoretical framework has been highlighted in section “Bubble model.” Section “Methodology” described the techniques employed in order to evaluate the analysis, while the data explanation and trends are explained in Sect. Data. The results of the study are discussed in section “Empirical analysis” and we have concluded the study in section “Conclusion and policy recommendation.”

## Literature review

In their study, Cadot et al. (2009) explain that VNL price witnessed a booming trend in the late 1990s and early 2000s. However, ever since then, the price declined rapidly because of the cyclones that were experienced in Madagascar. Moreover, the regulation of the Madagascar VNL market, in the wake of the pressure from the International Monetary Fund (IMF), leads to a highly volatile market as attributed toward the dramatic boom, followed by a significant perforation of the bubble that was created. At another instance, Laney and Turner (2015) observed that the economic liberalization policies led to the expansion of the VNL, but the internal production goals may have caused obstacles in the market participation. Then, Wexler (2017) also revealed that the VNL price increased due to the rising import bills pertaining to the USA, which have become double as compared to the previous year. Moreover, the frequent cyclones which destroy the VNL producing regions often lead to the shortage of products, which when unable to meet the required demand, push the price upward. At another time, Osterhoudt (2017) also analyzed that the VNL crop witnessed a low price as a result of the prolonged bust market, which encouraged the farmers around the globe to abandon VNL cultivation on a wide scale. Moreover, Zhu (2018) also indicated that the VNL price touched its highest level, after a decade-long period of being low. In this regard, Madagascar was still the region that held a dominating position in the global VNL production. In addition to this, Piling (2018) analyzed the real price boom of the VNL crop in Madagascar and concluded that the VNL price is primarily driven by speculation, poor harvest, and money laundering. This is what makes VNL as expensive as precious metals such as pure silver. Pagano and Echevarria (2018) also found that VNL price reaching their highest level, along with the VNL bean theft, complex pollination, and extreme weather are the prime determinants that have been responsible for the price hike. In the same context, in their study, Neimark et al. (2019) analyzed that the VNL price trends are suggestive of the insecurity and violence by the peasants, which then led to the price increase. Similarly, another valid reason for the price spikes occurred due to natural disasters such as cyclones, which were then followed by the rapid price crash of the VNL crop.

The price transmission has been explained in several studies in the extant literature. For instance, Meyer and von Cramon‐Taubadel (2004) concluded that contemporary literature emphasizes upon the method driven analysis and has little consideration toward the price transmission. Moreover, at another instance, Santeramo and von Cramon-Taubadel (2016) explained that for perishable products, the asymmetry of vertical price transmission also tends to disappear. In addition to this, Lence et al. (2018) found that the speed of price transmission tends to be unfair in all the time periods and the transfer cost is underestimated in a systematic fashion. Also, Goodwin et al. (2021) showed the presence of unit elasticity in the local market price transmission. von Cramon-Taubadel and Goodwin (2021) showed that the relationships between prices are strengthened due to the advances in marketing, information, and transportation technology. Moving on, the preceding literature comprises of studies that have made the use of different tests in order to investigate the bubble that develops in the asset prices. In this regard, Lucas (1978) coined that a bubble can be known as the deviation of the asset prices from the fundamental values. At a different time, Shiller (1988) also built upon the concept and developed a base in order to evaluate the bubbles through the variance bound test. However, the test that was developed is not particularly useful to cover the bubbles. For this, West (1987) then introduced a two-step methodology for the estimation of bubbles, which is now considered as the most important technique for bubble detection. However, the studies of Engle and Granger (1987); Narayan and Narayan (2007) employed the momentum threshold autoregressive (MTAR) method in order to examine the explosive behavior of the assets. Moreover, Hall et al. (1999), Lucey and O'Connor (2013) also referred to the Markov switching augmented Dickey–Fuller (MS-ADF) test for this purpose. The literature also shows that Diba and Grossman (1988); Wu and Xiao (2002) made use of the cointegration methods so as to notice the no bubble hypothesis. Also, Geraskin and Fantazzini (2013) referred to the log-periodic power law (LPPL) method in order to detect the bubbles.

The above-listed techniques have their respective drawbacks as well, which may cause unconvincing and unreliable outcomes. In this regard, Evans (1991) showed that if the probability of a bubble is non-negligible in nature, the unit root test is hardly able to detect the periodic collapse of the bubbles. In the same context, Charemza and Deadman (1995) proved that the unit root test is in fact not an appropriate measure for studying the multiplicative process as it comes with a randomly explosive behavior. Moreover, there is an element of unreliable detection due to which the beginning and end time points of the bubbles cannot be accurately identified. Moreover, Lammerding et al. (2013) also emphasized that the LPPL method can efficiently predict the critical time of a bubble, but the same cannot be tested. Zhang and Yao (2016) reported that when there are multiple bubbles in the period under consideration, then the MTAR test is only suitable for estimating the bubbles. Thus, a concern that can be raised about the validity and reliability of these techniques has been registered by many studies (Su et al. 2017). Researchers Homm and Breitung (2012) termed that the methodology suggested by Phillips et al. (2011) has a better performance record as compared to the traditional recursive practices that are usually undertaken for structural changes and for recognizing the bubble recognition algorithm. In the same context, the SADF technique is considered to be a recognized approach to observe the existence of bubbles and is particularly helpful if the sample contains a single bubble (Phillips et al. 2011). On the other hand, there is a probability that multiple bubbles can occur in the lengthy sample, and these are generally more complicated to detect when taking the econometric approach into account, primarily due to the weak detecting power of the existing method (Khan et al. 2020). The GSADF approach investigates the issue, so as to evaluate the occurrence of multiple bubbles. This approach has the benefit of allowing a changeable window width in the recursive regression which typically helps to increase the detecting power and dating methods (Khan et al. 2021). Likewise, it is more appropriate to examine any frequency data as well. Consequently, the method is more helpful than the previous methodologies in observing the existence of multiple bubbles. In order to eliminate these problems, it has been suggested in the extant literature that the SADF and GSADF tests be referred to. Therefore, the present article employs the SADF and GSADF tests to inspect VNL price bubbles.

## Bubble model

We have employed the bubble model to estimate the explosive behavior of the prices. It outlines that a bubble exists if the asset prices surpass the defined intrinsic values (Su et al. 2019a; Su et al. 2019b; Su et al. 2020a; Wang et al. 2020; Su et al. 2020b). A bubble occurs when the purchasers are prepared to pay more than the fundamental prices in order to buy the desired assets (Gürkaynak 2008). As per the model, VNL has been illustrated as follows.

1 $$P_{t} = P_{t}^{f} + b_{t}$$

where $P_{t}^{f}$ is the intrinsic price of coal and $b_{t}$ denotes the attributes of the bubble. In this regard, Eq. (1) represents the market fundamentals and the bubble. It must be noted that VNL depends on the market fundamentals when $b_{t} = 0$, while $P_{t}^{f}$ influences VNL when $b_{t} \ne 0$. The outcomes reveal multiple bubbles in the global VNL.

## Methodology

We can perceive a price bubble through the SADF test as proposed by Phillips et al. (2011). The right-hand unit root test based on the forward recursive regression has been employed in order to evaluate the recurring behavior of the unit root relative to the explosive price. It is noteworthy that The SADF tests inspect the consistent failure of the prices and subsequently the bursting of the anticipated price bubbles. Hence, The ADF and Phillips-Perron tests provide the basis for the stationarity tests with an explosive alternative as follows.

2 $$\Delta K_{t - 1} = \omega + \theta K_{t - 1} \mathop \sum \limits_{i = 0}^{l} \delta_{i} \Delta K_{t - 1} + \varepsilon_{i} ,\;\varepsilon_{i} \sim NID\left( {0,\sigma^{2} } \right)$$

where $K_{t - 1}$ is the VNL price, $\omega$ is constant, $l$ is the number of lags and *NID* denotes the normal and independent distribution. In addition to this, “The null hypothesis is $\theta = 1$, which means that the function $K_{t - 1}$ has a unit root test. Moreover, the alternative hypothesis $\theta > 1$ implies that $K_{t - 1}$ is explosive.” It is also affirmed that the opening location of the sequence is always 0; therefore, window size $g_{w }$ changes from $g_{0} \;{\text{to }}\;1.$ The end point $g_{2 }$ changes from $g_{0} {\text{to }}g_{1}$ therefore, the SADF statistic is as follows:

3 $$SADF_{{g_{0} }} = \sup_{{g_{{3 \in \left( {g_{0,} 1} \right)}} }} \left\{ {ADF_{{g_{2} }} } \right\}$$

where $g_{w }$ is the window size. For a single bubble, the SADF approach is deemed to be appropriate, however, while the VNL has multiple bubbles, the same approach is deemed to be unsuitable. However, Phillips et al. (2012, 2015) pointed out certain limitations of the SADF process in order to assess multiple bubbles. In this regard, the GSADF test flexible window explains this complication as well (Phillips et al. 2012, 2015). It repeatedly makes a series of sample systems that tend to emphasize on the appropriateness of the ADF test. For instance, this particular technique shifts the end of the regression $g_{2}$ from $g_{0}$ to 1 and expands the sample by shifting the initial and end points of the recursion in the adjustable window range to the viable range. In the same context, especially, the GDSAF permits the $g_{1}$ to alter from 0 to $g_{2} - g_{0}$. There seems to be an accurate identification of these multiple bubbles particularly after the addition of the greater windows and more data sub-samples. At two different instances, Phillips et al. (2012, 2015) considered the GSADF statistic to be the largest ADF statistic within a feasible domain of $g_{1}$ and $g_{2}$; therefore, this statistic portrayed as *SADF* ($g_{0}$) is displayed as follows:

4 $$SADF_{{g_{0} }} = \sup_{{g_{2} \in \left( {g_{0} ,1} \right)g_{1} \in \left( {0,g_{2} - g_{0} } \right)}} \left\{ {ADF_{{g_{1} }}^{{g_{2} }} } \right\}$$

Equation (5) shows the limit of the GSADF test, while adding the random walk and the null hypothesis in the regression model, which is demonstrated as follows:

5 $$\sup_{{g_{2} \in \left( {g_{0} ,1} \right)g_{1} \in \left( {0,g_{2} - g_{0} } \right)}} \left\{ {\frac{{\frac{1}{2}g_{w} [w (g_{2} )^{2} - w\left( {g_{1} )^{2} - g_{w} } \right] - \mathop \smallint \nolimits_{{g_{1} }}^{{g_{2} }} w\left( g \right)d_{g} [w\left( {g_{2} } \right) - w \left( {g_{1} } \right)}}{{g_{w}^{1/2} \left\{ {g_{w} } \right\}\mathop \smallint \nolimits_{{g_{1} }}^{{g_{2} }} w (g)^{2} d_{g} - \left[ {\mathop \smallint \nolimits_{{g_{1} }}^{{g_{2} }} w(g)^{2} d_{g} } \right]^{2} \}^{1/2} }}} \right\}$$

where is noted that the function $g_{w = } g_{2} - g_{1}$.

The method begins with the standard Wiener method, with constant and unsystematic features leading to sampling with a bounded point. Here, it is assumed that the functions $n_{1} ,n_{1} \ldots n_{N}$ have a fixed space between them, and the break is equivalent. “The Gaussian random variable with mean 0 and variance 1/N obtained at each point.” The critical value of the GSADF statistics exceeds the right-tail critical value of the SADF statistics. Therefore, we have evaluated the asymptotic critical values via numerical simulation, while the bootstrap method is a mean of the finite sample distribution. The process typically includes the data marking strategy, and therefore, it is irrational for it to be altered by the major motive for VNL feature explosion.

## Data

**Fig. 1:**
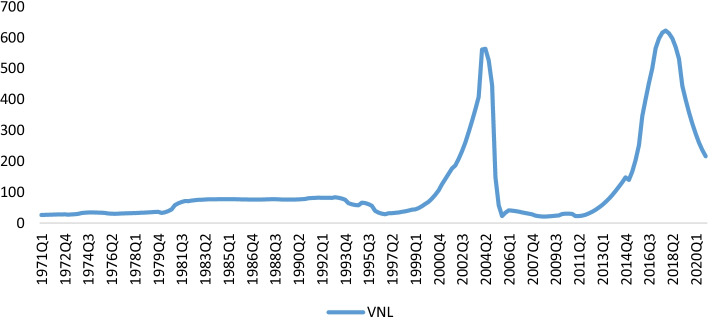
The trend of VNL price

When looking at the data, we have evaluated the quarterly data from 1971 to 2020 of the global VNL nominal price in order to analyze the potential bubble behavior. The data have been retrieved from the Nielsen Massey report^1^ which has been converted to a quarterly level data by the quadratic mean average method. It is also noteworthy here that the VNL market was typically characterized by corruption and opaque trade links in the 1970s, primarily because of political and economic interests (Neimark et al. 2019). It was the socialist revolution in Madagascar that led to rent-seeking, inefficiency, and corruption, which are considered to be the primary hallmarks of this time period. Similarly, the situation in other sub-Saharan countries has also not been suitable for VNL production. The farmers have only received less than 8% of the VNL price as a result of the export taxation. Moreover, Export quotas and licenses have been politically allocated, leading to market monopoly. Furthermore, countries such as Indonesia successfully entered the global VNL market, primarily driven by the cartels’ over-pricing strategy (Cadot et al. 2009). The new government in Madagascar regulated the VNL trade in order to stabilize the global supply and provide safety assurances for the farmers. It also introduced price regulation measures at the request of the International Monetary Agency in the 1990s, which then caused the prices to soar upward. Similarly, the government also initiated another episode of reforms in 1995, such as the elimination and stabilization of funds, abolishing export taxes and restricting government intervention. Then, in 1997, the government abolished the VNL fixed price policy, allowing prices to change with the market behavior, resulting in unprecedented price fluctuations. In this regard, Fig. 1 exhibits the VNL price behavior.

The price of VNL dropped at the end of the 1990s due to the increase in production, mainly from Madagascar and Indonesia, leading to an oversupply in the global market. Moreover, introducing the synthetic VNL at a cheap price has had a significant impact on the real VNL price. Likewise, the oil drilling and refining in VNL producing areas have also ruined the ecosystem, and therefore, the plantations have become more susceptible to plagues and diseases. The VNL price experienced a decline in the early 2000s to around 40 per kilogram mainly because of a cyclone that damaged the VNL crop in Madagascar. Also, the political crisis in 2002 deferred and hindered the VNL production in Madagascar, which had an extensive impact on the global VNL price. However, the VNL price reached a record high level of $230 per kilogram in the year 2003. The price also declined because the bumper VNL bean harvest in Madagascar, before collapsing to a price that was as low as $30 per kilogram. This tricky position proved to be critical for farmers in India and Indonesia primarily due to the production in Madagascar that projected to triple from 500 tons in 2003 to around 1,500 tons. Due to this, the VNL price fluctuated and remained as high as $500 per kilogram in the time period between 2003 and 2004. The VNL price remained at its highest levels between the years pertaining to 2001 and 2005, which were mainly caused by political issues and devastating storms. The price fluctuations were observed to be drastic as they went from $400 per kilogram in 2003 to $30 per kilogram in 2005, a situation that lasted for a decade. Meanwhile, the farmers abandoned the VNL crop cultivation in the year 2005, thus leading to a decade-long market depression between the years pertaining to 2005 and 2015. This prompted countries such as Uganda, India, Costa Rica and Colombia to enter the market and plant the first VNL crops in their respective areas. However, the rising production and falling demand from the food manufacturers thus led to the VNL prices to fall by 90%.

The VNL price experienced an increase in the year 2007, as five devastating hurricanes hit Madagascar and destroyed 25% of the crop. However, the VNL price declined to just $20 during the time span pertaining to the years 2008–2010. The VNL price dropped to the lowest level between the years 2005 and early 2014 due to the abundance of VNL production, while the production was at the highest level, and price was at a low level, respectively. The supply from other countries such as Uganda and India peaked in the year 2008, which then declined in the year 2014. The VNL price showed a long-bust period and the lowest price, and reason can be the production of vanillin, which is a close but artificial substitute for natural VNL. This has therefore reduced the demand for natural VNL, and the farmers in Madagascar have now given up the cultivation of natural VNL due to the low returns that they have experienced. Thus, the VNL price remained low between the years between 2005 and 2012, therefore, the health concerns arose due to the use of vanillin use, followed by the rising natural VNL usage in ice cream production in the year 2015. The demand exceeded the supply in the year 2015 for organic products, price speculation, and cyclone Enawo, which led to the wastage in the production regions specific to VNL. In more recent times, the price has increased relentlessly toward the highest level it has ever experienced, and hovers between $600 and $750 per kilogram due to the lower level of supply. Meanwhile, there was a price plunge in the year 2020, from the highest level achieved in the year 2018, as the food service industry had been affected by the coronavirus (COVID-19). However, industrial VNL has benefited from the novel COVID-19 as people started preparing their meals at home. In this regard, Table 1 highlights the summary statistics of the VNL price $per kg during the considered time period. As per the information, there seems to be a tremendous difference between the maximum and minimum values which indicate price instability. It implies that the price of VNL has had successive ups and downs over the sample period, which has been confirmed by the observed behavior. As seen in Fig. 1, the behavior exhibits a boom-and-bust period, as driven by various factors. Similarly, the standard deviation reveals greater VNL price fluctuations. The skewness is also positive and shows that the price is skewed toward the right. Moreover, the VNL price is leptokurtic distributed, as the value is greater than 3. However, the series is non-normally distributed, as per the Jarque–Bera test.

**Table 1:** Descriptive Statistics

Variables	Mean	Maximum	Minimum	SD	Skewness	Kurtosis	J–B
VNL pric price	116.544	622.650	20.909	146.443	2.187	6.756	277.098***

***is the significance level at 1%

VNL price is $per kg

## Empirical analysis

**Fig. 2:**
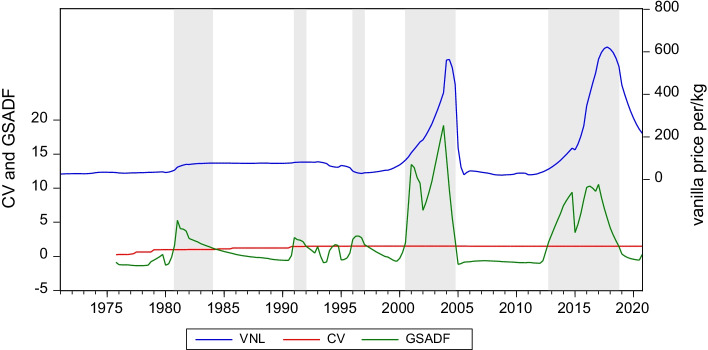
GSADF test of VNL price

Table 2 illustrates the result of the twin tests such as the SADF and GSADF, for examining the explosive behavior of the VNL price. The underlying null hypothesis is thus rejected, which confirms the existence of an explosive nature in the VNL price. Thus, the results provide a reason to detect the potential bubbles in the VNL price. We have also employed the GSADF procedure for VNL price bubble evaluation, which is presented in Fig. 2. The graph comprises of three lines, one in the middle representing the GSADF statistics with a 95%, and one at the lowest representing test statistics (Khan et al. 2020). Moreover, the complete sampling period displays five VNL price bubbles, including the start and the end of the bubbles. In this regard, Phillips et al. (2015) argue that the GSADF test tends to have a better performance as compared to the SADF tests and covers more data in the sub-sample data. The results offer further facts on the causes of the bubble factor.

**Table 2:** The SADF and GSADF tests

VNL price	SADF	GSADF
	16.971***	19.201***
**Critical value**		
90%	0.938	1.905
95%	1.168	2.247
99%	1.662	2.721

***shows significance at the 1% level

The data show that there was a detection of bubbles around 1980, 1990, 1996, 2001 and 2014, as illustrated in Fig. 2. The length of these bubbles indicates that the VNL price experiences short- and long-term bubbles. That is to say that the bubble detected during the time periods pertaining to 1990–1991, 1996–1997 is short term, as it appears, and these bubbles have burst in the same year as well. However, the bubble detects that the time spans pertaining to 1980–1983, 2001–2004 and 2014–2018 are termed to be long term primarily because the existence of these bubbles occurs over the years. As observed, the first bubble started in 1980Q4 and ended in 1983Q4. It is also observed that the VNL price increased sharply in the year 1980 despite there being the introduction of the Indonesian variety of VNL due to the Tropical Cyclone Hyacinthe, which ravaged the cropland. This resulted in a large drop in the VNL production, which was caused by the rising prices, and translated into supply shortages (Menz and Fleming 1989). Similarly, the VNL supply was dominated by a cartel (Univanille) that colluded with Madagascar, Reunion, and Comoros Islands, which supplied three-quarters of the world VNL (Laney and Turner 2015). This cartel has controlled VNL price and distribution, which kept the exports low and international prices extremely high, therefore earning high taxes (Melo et al. 2000). Meanwhile, the VNL market was organized by the marketing board, CAVAGI (Caisse de Commercialisation et de Stablilisation des Prix du Café) which kept the prices relatively low (Krivonos 2004). In this era, the price was around $70 per kilogram because of the manipulation of the Univanille (Menz and Fleming, 1989). However, the International Monetary Fund (IMF) pushed Madagascar to liberalize its economy in the mid-1908s and introduce various policies which would have a significant impact on the VNL prices (Laney and Turner 2015) in the meantime, though, the price of VNL experienced a burst and declined by 70% and remained around 20 per kilogram.

The second bubble started in the year 1990Q4 and ended in the year 1991Q4. The alliance of political and economic interests appeared in the vanilla market in the socialist regime. This distressed the vanilla trade and global supplies due to greater government intervention, which made the farmers insecure. Thus, the market became deregulated during this period, particularly at the behest of the international monetary organizations, which produced higher volatility in the market and followed the trend of the rising VNL price (Neimark et al. 2019). The bubble burst when the new policy was implemented. As per the new policy, the farmers earned lesser amounts of money due to the export taxation and exports licenses which were unfairly allocated on a political basis. Similarly, cooperative management was replaced by a centralized system which led to negative consequences for the vanilla market. Eventually, the export taxes experienced a spike of to 82%, while the prices that the farmers received were quite low. Thus naturally, the low prices discouraged the farmers from cultivating more VNL, which resulted in low production (Melo et al. 2000).

The third bubble started in 1996Q1 and ended in 1997Q1. A remarkable rise in the VNL price was witnessed and new competitors such as China started entering the market. Although China invested $16 million to the cause, but it remained relatively unsuccessful at growing the VNL beans. During the time period, the market was controlled by the Madagascar government and farmers received a small price, while the export price and government earnings were on the higher side. The market-based price policy was also introduced to minimize the government intervention in this regard. In addition to this, the CAVAGI initiative was abolished as well, along with the licensing system, which resulted in the sharp reduction in the global VNL prices (Maret 2007). Likewise, the fixed price for producers was gradually lifted which was responsible for weakening the global VNL price. The free trade system for VNL was then implemented on the directive of the EU and the World Bank, while the regulated plan system was abolished, which was reflected in the falling prices of VNL. Similarly, the exchange rate correction in several sub-Saharan African countries resulted in currency overvaluation, which also discouraged the farmers to renew and maintain their crop quality (Melo et al. 2000). Moreover, the export tax and other tools of government control were completely abolished in 1997, but still the VNL sector did not recover as expected (Maret 2007).

The fourth bubble started in 2001Q1 and ended in 2004Q4. During this period, the VNL price rose sharply because the cyclone Hudah hit Madagascar and damaged the VNL crop, which may have caused a disruption in the supply. However, the annual VNL bean grew by 30% and the price rose to $500 per kilogram in the year 2003 (Laney et al. 2015). Furthermore, the political instability, natural disasters such as cyclones and poor weather drove the VNL price up in the year 2004. This over-pricing attracted new countries into the VNL market. Due to these factors, the prices reached a record level, while and imports remained at the lowest level due to the unfavorable weather conditions and crop theft issues in Madagascar. Moreover, the price of natural VNL was definitely affected by demand due to the fact that the price of real VNL is already very high, so therefore, to maximize their profits, the companies continued to increase the use of synthetic VNL (vanillin) in their products. This trend was observed in 2003 when the VNL price reached a record level. The highest level prompted the VNL importers to actively seek VNL substitutes, while also exploring further technological advancements in synthetic VNL production. Therefore, the end resulting product called vanillin was a product which proved to be an important factor in real VNL price changes, and its biggest competitor. The reason for this was that the production cost, to this day, is extremely low and accounts for more than half of the VNL market. It is noteworthy that the VNL price bubble then burst in the year 2004 due to the bumper VNL crop, and decreasing demand caused by the production of vanillin. This then pushed the VNL price down to $40 per kilogram in the year 2005. The high price of VNL prompted global competitors to enter the market, while the consumers switched to cheaper artificial VNL, which may have caused the decline in the price that followed (Laney et al. 2015). Similarly, the VNL price fell sharply in the year 2004, mainly due to excessive harvest of about 1,500 metric tons, causing ample supply in the market.

Finally, the last bubble started in 2014Q3 and burst in 2018Q3. During this time, the VNL price spiked at an extraordinary rate and remained at a staggering $400 and $600 per kilogram, thus driven by higher global demand and a decrease in the supply as experienced in the previous bubbles, the price speculation and the cyclone contributed to the exorbitant price increase. It was noticed that the trend of the rising prices was coinciding with the demand for VNL from multinational companies as they abandoned vanillin and started preferring the real one, which may have reflected in the price hikes (Laney et al. 2015). Then, it was observed that the VNL production declined to 1200 tons in the year 2015, as compared to 1800 tons of the previous year, whereas a high demand experienced simultaneously resulted in the abnormal rise in the price. That is to say that during this time, the price was recorded to $400 per kilogram. The main factors contributing to this were the cyclones that damaged the VNL crop, while at the same time, the demand was strong. Moreover, the commodity speculation by large buyers forced the prices to hike upward (Neimark et al. 2019). Therefore, it was then observed that the VNL prices reached a record high level of $600 per kilogram in May 2018 and made it a commodity that was more expensive than silver. The VNL price then continued to spike since the year 2017 mainly due to the cyclone Enawo, which devastated the VNL crop and inflicted a $400 million damage to the industry. Moreover, a rise in theft also impacted the farmers and smugglers who had bought considerable amounts of VNL in order to launder their money (Osterhoudt 2017). The use of the VNL market to launder money from illegal sales of rosewood tends to trigger many of the price spikes. Similarly, the decline in the quality of the crop is another reason for the soaring prices, as the crop had been harvested before it even matured. This was primarily due to the farmers’ concerns regarding theft and the belief that the prices may fall before the harvest season. However, the early harvest led to a compromise on the quality of the crop, as it was quite low. At the same time, the rising price decreased the demand for real VNL, and the producers increased their production of vanillin. After this, the farmers reacted by storing their VNL crop, only to go on and earn $800 per kilogram, which again drove up the price further and the buyers paid ridiculously high amounts of money to fulfil their commitments with international food companies that were demanding VNL (Neimark et al. 2019). The VNL price bubble burst in the last quarter of 2018, followed by the decline in the demand for real VNL, and an increase in the demand for vanillin, as the real VNL retailed at a price that was as high as $500 per kilogram. This high price of VNL pushed the big companies to redirect their use toward vanillin, that too at a low cost, which resulted in a natural decline of demand for the real VNL. Similarly, a larger crop was being produced in other regions such as Papua New Guinea, Indonesia, and Tahiti, further causing the price to fall by 50%. Importers started seeking VNL producing areas other than Madagascar, which has been considered as the gold standard for VNL. Meanwhile, the Madagascar government introduced different prices for different quantities of VNL and hoped that setting a minimum price would help then to regulate the market (Cadot et al. 2009; Zhang and Yao 2016). In this regard, the minimum price for the lowest quality, and the higher quality product to trade at a premium price were introduced. However, this policy had the opposite effect, as the highest quality products were traded at the lowest level, and buyers were not prepared to pay a premium. Therefore, the lowest and the highest quality products were sold at the same price.

## Conclusion and policy recommendation

This paper examines the bubbles that were experienced in the global VNL price by employing the GSADF approach. The result showed that there were five bubbles in the VNL price driven market, due to very specific reasons. The unfortunate advent of devastating cyclone and the monopoly of the cartels caused the first bubble. Similarly, the second bubble was driven by price regulations and export taxation, which exerted negative consequences for the VNL price. However, the third bubble resulted from introducing a market-based policy, abolishing the cartels, and exchange rate correction. Political instability, devastating cyclones and poor weather mainly drove the fourth bubble. The last bubble was caused by a higher global demand and decreased supply. Likewise, the price speculation and the cyclones also significantly contributed to the price increase. Moreover, the rising price also coincided when the multinational companies abandoned synthetic VNL and started preferring the real one, which may have reflected in price fluctuations. Also, the decline in the quality of the harvest was another reason for the soaring prices of VNL.

This study offers useful policy suggestions and implications for the concerned stakeholders and governments. First, the results show that the VNL price is highly susceptible to changes in supply, uncertain weather conditions, and speculation. These factors on the supply side are the main contributors to the fluctuation of the VNL price. Thus, greater stability is essential to the VNL market for continuous supply, which can lead to the total control over the price volatility that has thus far been experienced. Then, the balance between the demand and supply, and the monitoring role of the government in price stability proves to be extremely necessary in this regard. Second, the middlemen have been exploiting the farmers and contributing significantly toward the spikes in the price. This manipulation has typically led to a decline in VNL cultivation, mainly because the farmers have experienced low returns on their crops. Similarly, the farmers switched to other crops, which may have caused falling production which ultimately led to price instability. Continuing with the same stride, it can be observed that the power to determine the ultimate power lies with the buyers in the VNL market, while the individual farmers have no say in the price highs and lows, and the middlemen control the prices. Thus, abolishing the cartels and middle role was an extremely vital steps toward the exorbitantly high price phenomenon which then attracted more farmers to the VNL cultivation and increased the production as well. Moreover, in this regard, it is suggested that the governments should facilitate the big companies to directly negotiate with the framers, a step which may be beneficial for both companies and the farmers. Third, the problem of theft and low quality of VNL led to violent price fluctuations. The farmers harvested their crops before it matured, primarily because of the theft which had previously led to low-quality VNL. This eventually led to a decline in the price. Thus, in such a scenario, in idea terms, the government should have taken the appropriate measures to overcome the theft problem and could also form a separate force for such security. Again ideally, the government should have enacted special laws regarding the early harvest of the VNL crop, and levy strict penalties for early harvesting. Lastly, the market structure may have contributed toward the price fluctuations. This is mainly because on typical terms, the market liberalization follows unprecedented price volatility and huge fluctuations often lead to price speculation and unpredictability regarding the price and the quality of VNL. Thus, stability is an important component for all the stakeholders in the market. Similarly, the market structure should be devised in such a way that involves all the participants, to avoid any factors pertaining to instability.

## Data Availability

Data can be provided on a request.

## Abbreviations

VNL: Vanilla
EU: European Union
SADF: Supremum Augmented Dickey–Fuller
GSADF: Generalized Supremum Augmented Dickey–Fuller
IMF: International Monetary Fund
MS-ADF: Markov switching augmented Dickey–Fuller
LPPL: Log-periodic power law
MTAR: Momentum threshold autoregressive
CAVAGI: Caisse de Commercialisation et de Stablilisation des Prix du Café

## Footnotes

1 https://nielsenmassey.com/
